# Memory-efficient low-compute segmentation algorithms for bladder-monitoring smart ultrasound devices

**DOI:** 10.1038/s41598-023-42000-9

**Published:** 2023-09-30

**Authors:** Zhiye Song, Mercy Asiedu, Shuhang Wang, Qian Li, Arinc Ozturk, Vipasha Mittal, Scott Schoen, Srinath Ramaswamy, Theodore T. Pierce, Anthony E. Samir, Yonina C. Eldar, Anantha Chandrakasan, Viksit Kumar

**Affiliations:** 1 Department of Electrical Engineering and Computer Science, Massachusetts Institute of Technology, Cambridge, MA USA; 2 Center for Ultrasound Research and Translation, Massachusetts General Hospital, Boston, MA USA; 3 Texas Instruments, Dallas, TX USA; 4 Department of Computer Science and Applied Mathematics, Weizmann institute of Science, Rehovot, Israel; 5 Department of Ultrasound, Shenzhen University General Hospital, Shenzhen, Guangdong China

**Keywords:** Ultrasonography, Biomedical engineering, Bladder, Computer science, Computational science

## Abstract

Post-operative urinary retention is a medical condition where patients cannot urinate despite having a full bladder. Ultrasound imaging of the bladder is used to estimate urine volume for early diagnosis and management of urine retention. Moreover, the use of bladder ultrasound can reduce the need for an indwelling urinary catheter and the risk of catheter-associated urinary tract infection. Wearable ultrasound devices combined with machine-learning based bladder volume estimation algorithms reduce the burdens of nurses in hospital settings and improve outpatient care. However, existing algorithms are memory and computation intensive, thereby demanding the use of expensive GPUs. In this paper, we develop and validate a low-compute memory-efficient deep learning model for accurate bladder region segmentation and urine volume calculation. B-mode ultrasound bladder images of 360 patients were divided into training and validation sets; another 74 patients were used as the test dataset. Our 1-bit quantized models with 4-bits and 6-bits skip connections achieved an accuracy within $3.8\%$ and $2.6\%$, respectively, of a full precision state-of-the-art neural network (NN) without any floating-point operations and with an $11.5\times$ and $9.0\times$ reduction in memory requirements to fit under 150 kB. The means and standard deviations of the volume estimation errors, relative to estimates from ground-truth clinician annotations, were $5.0\pm 33$ ml and $6.8\pm 29$ ml, respectively. This lightweight NN can be easily integrated on the wearable ultrasound device for automated and continuous monitoring of urine volume. Our approach can potentially be extended to other clinical applications, such as monitoring blood pressure and fetal heart rate.

## Introduction

### Wearable ultrasound devices for continuous monitoring

Miniaturization of ultrasound devices enables smart medical imaging devices, i.e., wearable devices that collect and process medical images in real time^1,2^. Since any tissue amenable to conventional diagnostic ultrasound imaging may benefit from the capability of longitudinal monitoring, smart medical imaging devices have many clinical applications, including blood pressure monitoring, neuromodulation and bladder volume monitoring^3–5^. In blood pressure monitoring, segmenting the blood vessel in consecutive ultrasound image frames and calculating the blood pressure from its diameter avoids the discomfort from cuff inflation in oscillometric measurement^3,6^. In another application, Pashaei et al.^4^ segmented the vagus nerve in the ultrasound image, and used the segmentation to guide ultrasound beams in neural therapy.

**Figure 1:**
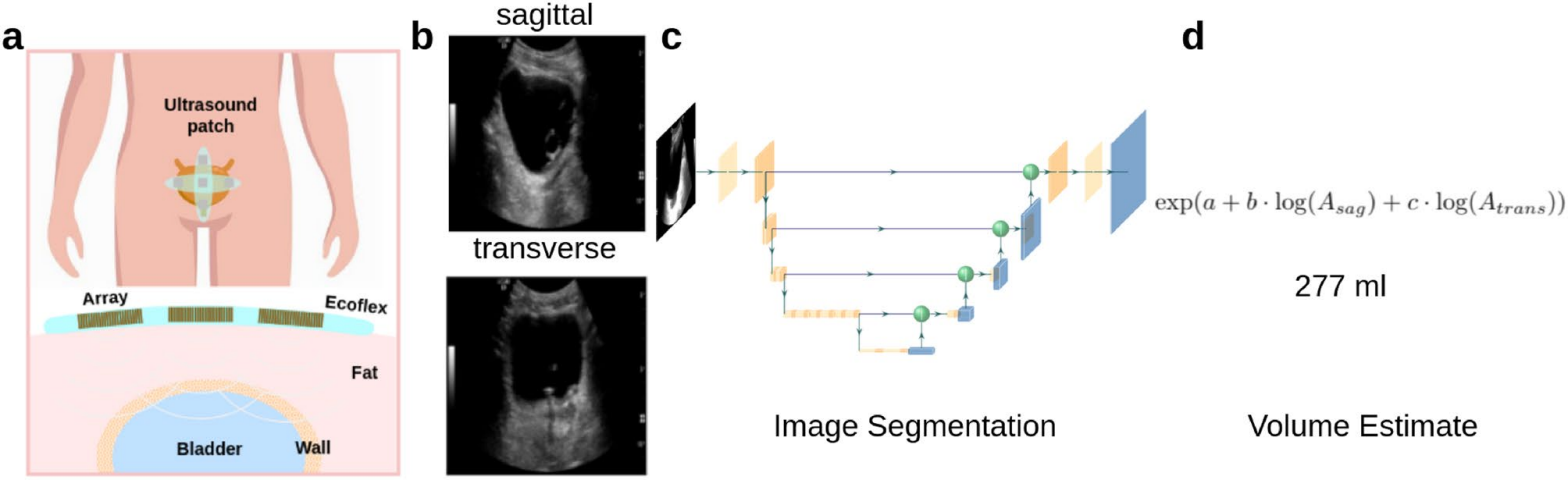
Proposed clinical workflow of bladder patch device and volume estimation. (**a**) (**b**) An ultrasound patch collects images. (**c**) A lightweight ML segmentation algorithm that runs on an ASIC in the wearable device. (**d**) The bladder volume is calculated.

Ultrasound imaging is currently used to estimate bladder volume due to its portability, non-ionizing radiation, and low cost. The bladder volume can be estimated from a segmented bladder mask in the ultrasound image^7,8^ (see Fig. 1). However, current approaches utilizing ultrasound imaging are limited for continuous monitoring due to the following: (1) for conventional handheld ultrasound devices, measurement must be performed by an operator, making continuous monitoring unfeasible; (2) commercially available continuous monitoring devices measure a few depth profiles instead of forming a full image, which compromises volume estimation accuracy for power efficiency; and (3) current point of care ultrasound systems are not sufficiently energy efficient to permit wearable device deployment.

Continuous bladder volume monitoring helps to diagnose incontinence or urine retention and reduces the need for indwelling urinary catheters^9^. For instance, up to $70\%$ of surgery patients experience post-operative urinary retention (POUR), the inability to urinate after a surgical procedure despite having a full bladder^10^. It may be accompanied by discomfort, bladder spasms, and urine leakage. It may also progress to renal obstruction, acute and chronic kidney injury, and ultimately renal failure. POUR can be easily managed and reversed via intermittent or indwelling urinary catheterization once diagnosed. However, in about $60\%$ of patients, symptoms can be masked and go unnoticed due to anesthesia, analgesia, or insensitivity related to chronicity of obstruction, leading to poor post-operative outcomes, urinary tract infections, prolonged bladder overdistension, and increased length of hospital stay^10^. With continuous bladder monitoring, POUR can be diagnosed in its early stage and prevent future complications.

In the context of long-term care facilities, 7–10% of residents have urinary catheters^11^. However, the management strategy of aggressive bladder drainage via catheterization is not optimal due to (1) poor patient acceptance and (2) a high rate of complications, such as catheter-associated unitary tract infection (CAUTI). In fact, a wealth of literature on the risks of indwelling catheters in hospitalized patients has led to national efforts to reduce urinary catheter utilization^12^. Continuous monitoring of urinary bladder volume could ensure that newly developed urinary retention is never missed and facilitate early catheter removal. In fact, it may form an important pillar to reduce CAUTI as advocated by the Agency for Healthcare Research and Quality^12^.

To overcome the limitations of current ultrasound systems and enable real-time volume monitoring, we developed an energy-efficient image segmentation algorithm that can be coupled with existing body contour ultrasound patches^1,2^. This algorithm enables continuous, automatic, and accurate bladder volume estimates in real time from images captured with the wearable ultrasound patch. Figure 1 outlines the device operation: (a) ultrasound patch collects ultrasound images in (b), (c) segmenting the bladder region in transverse and sagittal planes, (d) calculating the area of the segmented regions, and using the area as input to the double area method to estimate the urine volume^7,8^. To enable edge employment of real-time segmentation without a significant increase in energy or cost, the algorithm should not require floating-point support and only use minimal memory and compute resources.

### Neural networks in medical image segmentation

Recently, neural networks (NNs) have gained popularity for various medical image segmentation applications, including ultrasound imaging. Some commonly used architectures for medical imaging are U-Net, Panoptic, and DeepLabv3+. U-Net^13^, with the encoder-decoder structure, is the most popular segmentation architecture, as shown in past medical image segmentation works^14,15^. Panoptic Feature Pyramid Networks (FPNs) were initially designed for unifying instance segmentation and semantic segmentation; nonetheless, they are lightweight and yield high accuracy for semantic segmentation^16^. DeepLab3+ uses spatial pyramid pooling to extract contextual information at multiple scales^17^.

Since segmentation networks typically have billions of computations and millions of parameters, previous works have focused on quantization in convolutional layers. AskariHemmat et al.^18^ used 4-bit weight and 6-bit input for most convolutional layers in U-Net. TernaryNet proposed using the ternary quantization function as the activation function and used a continuous adaptation of tanh in back-propagation, achieving a mostly ternarized U-Net^19^. MedQ used extensive parameter-free skip connections, tanh in back-propagation, and distributional loss to achieve binarization and ternarization of most convolutional layers in U-Net^20^. Our previous work used skip connections and distribution learning to binarize U-Net, Panoptic FPN, and DeepLab3+^5^.

While these works significantly reduce the network size, they are still unsuitable for low-power, low-cost edge deployment for several reasons. First, the skip connections between the encoder and the decoder branches are floating-point (FP) feature maps^5,18, 20^. Since each FP value requires 32 bits, hundreds of thousands of the intermediate values require either a large on-chip Static Random-Access Memory (SRAM) or external access to Dynamic Random-Access Memory (DRAM). The on-chip SRAM dominates the chip area, thereby increasing ASIC cost^21^; external DRAM chip also increases product cost. Second, floating-point calculations are still needed for the first convolutional layers, BatchNorm layers, and skip connections (details in Supplementary Table S1). An FP addition consumes $30\times$ more energy than an 8-bit fixed-point addition, while an FP multiply is $5.5\times$ as energy-consuming as an 8-bit fixed-point multiply^22^. Lastly, while latency is not a concern in bladder monitoring, other real-time tissue monitoring tasks, such as cardiac motion, have stricter latency requirements^23^. Lowering the computation requirements and the number of memory accesses will make the algorithm run faster, satisfying the real-time requirement of an autonomous ultrasound monitoring device. Therefore, given wearable medical devices’ chip area and energy constraints, we require a fully quantized network with low-bitwidth skip connections and no floating-point operations.

### Contribution of this work

To address the challenge of deploying resource-hungry NNs on smart medical devices, we present and validate a power and memory efficient deep learning model for accurately segmenting the bladder region and calculating urine volume. We train our model on bladder images captured with a clinical ultrasound system. Multiple models with varying precision are proposed, allowing for a trade-off among accuracy, memory and power requirements. While calculating bladder volume is presented as the target application, this work has the potential to serve as a platform technology, deployable across a wide range of wearable, health-monitoring device applications that require accurate, real-time, autonomous monitoring on the edge.

## Methods

### Data collection and curation

A dataset of bladder ultrasound images was collected retrospectively from 1034 patients undergoing various bladder imaging procedures at the Massachusetts General Hospital (MA, USA) between February 2018 and October 2018. The Mass General Brigham (MGB) Institutional Review Board (IRB) approved the study as protocol #2020P001599; the requirement for informed consent was waived as per the MGB IRB. All methods were performed in accordance with the relevant guidelines and regulations including the Declaration of Helsinki. Four hundred and thirty four patients were randomly selected from the retrospective database of ultrasound bladder images, and bladder images were from the sagittal and transverse views. Images in which the bladder wall could not be visualized properly due to poor image quality were removed. Images were gathered using curvilinear probes on GE (GE Healthcare, Chicago, IL) and Philips (Philips, Amsterdam, the Netherlands) ultrasound systems. The dataset was annotated by five physicians with between 5 and 10 years of experience in bladder ultrasound interpretation. There is subjectivity in image annotation, especially considering that each image is annotated by one physician rather than a panel of physicians. The inner and outer bladder walls were annotated using a DICOM viewing software (MicroDicom, Sofia, Bulgaria). Annotation masks were extracted from the DICOM images using Python software, and both the masks and DICOM images were de-identified.

Images were down-sampled to 224 $\times$ 224 before feeding to the neural network. The physical scales were extracted from the DICOM images and used in the volume calculation. This pre-processing can be avoided in an integrated portable or wearable device as the beam forming grid can be adjusted to produce the required input size.

### Network architecture

#### Baseline architectures

Deep neural networks for image segmentation typically have an encoder-decoder structure. The encoder extracts features from the input image at different resolutions and fields of view. The decoder aggregates these features to generate a segmentation mask. Most NNs used for segmentation are variations in the encoder-decoder structure. Specifically, three main variants were explored to analyze the performance of the binarization and quantization techniques: (1) U-Net^13^ with a lightweight decoder, (2) Panoptic Feature Pyramid Networks with the output merging information from all decoder layers^16^, and (3) DeepLabv3+ with spatial feature pooling^17^. In their baseline full precision format, these networks have billions of floating-point operations (FLOPS) and millions of network parameters^16,20^, which are extremely high for low-power wearable applications. As an efficient alternative to these networks, Brahma et al.^5^ used depthwise separable convolutions, binarization of most convolutional layers with distributed learning, and real-valued parameter-free skip connections to maintain accuracy. The models in Brahma et al. were used as the baseline in this paper.

#### Network quantization

The B+FP Model used RSign and PReLU to binarize convolution layers (except the first layer), similar to Brahma et al. and ReActNet^5,24^. This quantization method is more robust to distribution shift of activations than the non-parametrized alternatives^24^. In the binary convolution, activations, *x*, were binarized with an RSign function, where $\alpha ^{fp}$ is an FP threshold parameter. The superscript indicates whether the value is FP or binary, and the subscript *i* is the channel number:

1 $$\begin{aligned} x_i^b= {\left\{ \begin{array}{ll} 1 &{}x_i^{fp}>\alpha _i^{fp}\\ -1 &{}x_i^{fp}\le \alpha _i^{fp}. \end{array}\right. } \end{aligned}$$

Similarly, the weight tensor, *W*, was binarized per channel using

2 $$\begin{aligned} W^B= {\left\{ \begin{array}{ll} 1 &{} W^{FP}>mean(W^{FP})\\ -1 &{}W^{FP}\le mean(W^{FP}). \end{array}\right. } \end{aligned}$$

ReLU was replaced with PReLU, with three trainable parameters, $\beta _i$, $\gamma _i$, $\eta _i$:

3 $$\begin{aligned} PReLU(x_i)= {\left\{ \begin{array}{ll} (x_i-\gamma _i)+\eta _i &{}x_i>\gamma _i\\ \beta _i(x_i-\gamma _i)+\eta _i &{}x_i\le \gamma _i. \end{array}\right. } \end{aligned}$$

**Figure 2:**
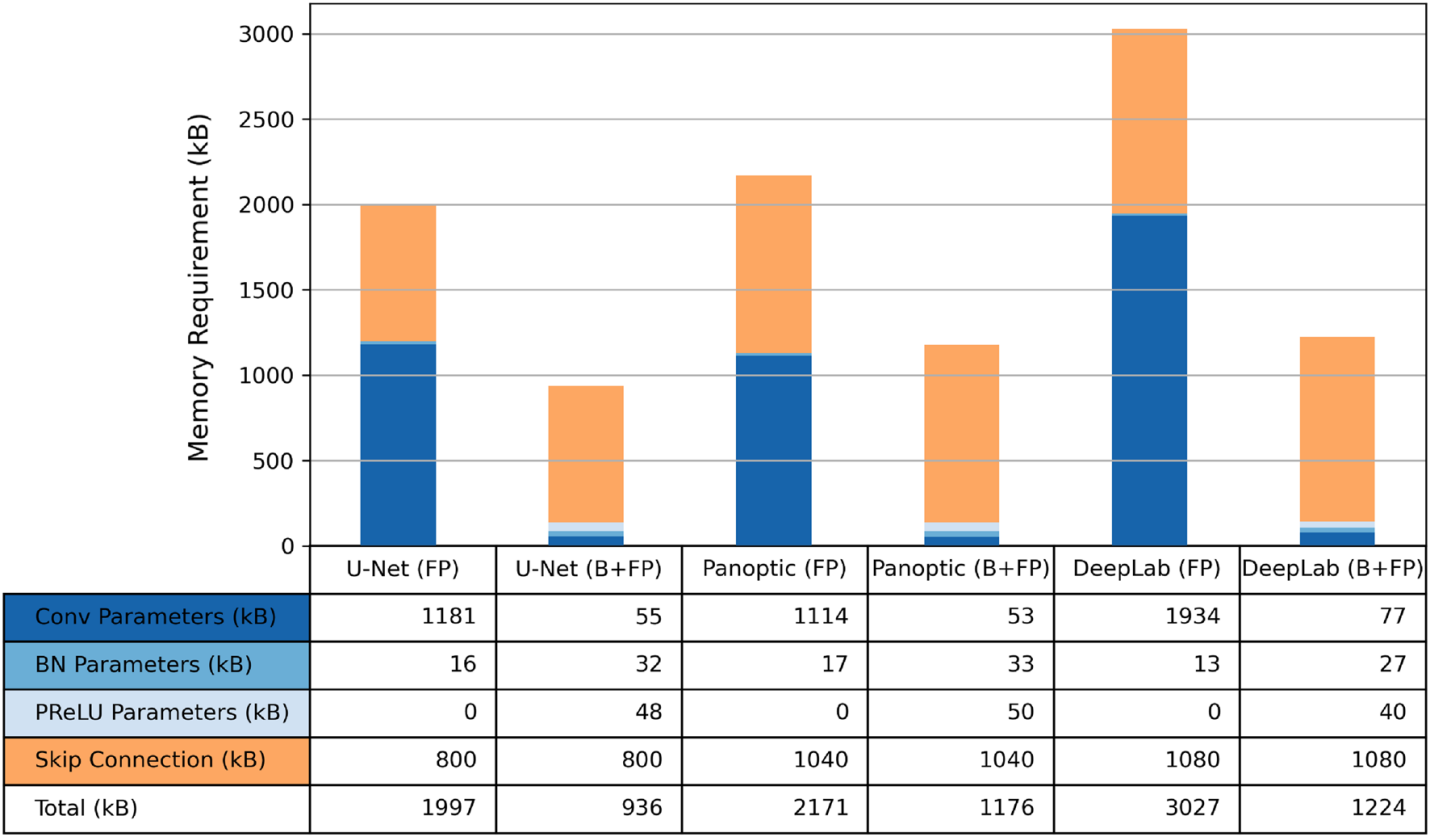
Memory requirement of FP and B+FP Models. Each column corresponding to an FP Model is followed by the B+FP model of the same algorithm. The B+FP Models typically consume half the memory of the FP Models. The U-Net has the lowest memory requirements of all three algorithms.

We proposed a new B+Q Model, where all the remaining FP modules were quantized as in Table 1. The number of bits for each parameter type was determined by its value distribution. A binary shift value, $s=clamp(round(\log _{2}(|w|)))$, replaced the multiplication by *w* required in BatchNorm (BN). The sign of *w* was absorbed by the binary weight tensor in the Conv layer immediately preceding the BN layer. As shown in Bi-Real Net^25^, floating-point skip connections greatly enhanced the performance of binary networks. However, skip connections were the most memory-intensive component during inference, as shown in Fig. 2. Therefore, experiments were performed to determine the skip connection bitwidth with the optimal trade-off between memory requirement and accuracy. Since each skip connection had a different range, a binary shift parameter was used for each skip connection.

**Table 1:** Layer precision for each model version (FP = floating-point; B = binary; Q = quantized).

Model version	FP model	B+FP model	B+Q model
Most conv layers	FP	Binary	Binary
First layer	FP	FP	4-bit input + 4-bit weight
Skip connections	FP	FP	4-bit/6-bit with scalable range per connection
BatchNorm	FP	FP	Binary shift + 4-bit bias
Activation function	ReLU	PReLU with FP $\beta$, $\gamma$ & $\eta$	PReLU with 2-bit $\beta$ and 4-bit $\gamma$ & $\eta$
RSign	–	FP threshold ($\alpha$)	4-bit threshold ($\alpha$)

#### Skip connection compression

After skip connections are quantized, they are easier to compress. This paper evaluates a variety of lightweight compression schemes in this context. Using the intermediate feature maps of the test dataset, the worst-case memory requirement of the compressed data was recorded. Zero-value compression (ZVC) includes a bit stream that indicates whether a value is zero, followed by all non-zero values. Differential ZVC (DZVC) takes advantage of the spatial correlation in an imaging application: a bit stream indicates whether the difference with the previous value is zero, followed by the new values. Bit-plane compression (BPC) is a more complicated compression scheme based on Kim et al.^26^. Both block sizes of 16 and 8 were explored to adapt to our applications, and the encodings of 01 and 001 were exchanged. The fourth compression method is an enhanced version of BPC (EBPC), which consists of ZVC followed by BPC^27^.

The threshold of the binary convolutional layers was quantized to a 4-bit signed integer of a fixed range, which was incompatible with the skip connection number representation. Instead of increasing the number of bits of the skip connections to achieve compatibility, storing the binarized skip connections separately preserved the accuracy with minimal memory overhead.

#### Inner and outer bladder wall annotations

Although bladder volume calculation only required the inner bladder wall segmentation, it was hypothesized that providing the outer bladder wall annotation would help the network extract imaging features better during training. To test this hypothesis, two identical NN models were trained, except that one had a one-channel output and the other had a two-channel output.

The one-channel output model only included the inner bladder wall, whereas the two-channel output model used inner and outer bladder walls. For two-channel output, the average Dice score of both output maps was used as the cost function. Whereas, during inference, only the filter corresponding to the inner bladder wall was used for volume estimation, as the urine volume only depends on the inner bladder wall. The two models’ Dice scores of the inner bladder wall were compared to test the hypothesis.

### Algorithm training and hyper-parameters

The bladder dataset consisted of 741 sagittal and transverse images from 434 patients: 473 of these images were used to train the network, 120 were used as the validation dataset for tuning the hyperparameters, and the test dataset consisted of 74 pairs of sagittal and transverse images. The training set was augmented by applying affine transformations (horizontal flips, shearing, and rotation by random angles) to the images during each training epoch.

The models were trained in PyTorch with custom backpropagation functions. We used the Dice score as the loss function. Cosine annealing scheduling policy with warm restarts with an initial learning rate of 0.005 was used for training. A weight decay of $4\times 10^{-5}$ was used in the FP Model training as regularization; no weight decay was used for B+FP or B+Q Models.

The B+Q Model was trained in 2 steps. First, we trained the B+FP Model from scratch with 150 epochs. The learned B+FP model parameters were used to initialize the B+Q network, and additional 150 epochs were performed. The model with the highest validation accuracy among the epochs was used.

Since the binarization function was not differentiable, a differentiable surrogate was required in backpropagation. For both the B+FP and the B+Q models, the standard Straight-Through Estimator (STE) and the hyperbolic tangent ($\tanh (2x)$^20^) were experimented as the surrogate. As shown in Supplementary Fig. S1, the B+Q Model with STE converged to a more optimal solution. Therefore, all models reported in this paper were trained with STE.

### Volume estimation

The pixels in the segmented bladder area of both sagittal and transverse views were counted separately. The bladder areas were converted from pixel counts to square centimeters using the physical scale information from the DICOM files, and then used in the formula of the double area method^7,8^:

4 $$\begin{aligned} V = \exp {\left[ 0.8304 + 0.5625\times \log {(A_{1}/1~\textrm{cm}^2)} + 0.7211\times \log {(A_{2}/1~\textrm{cm}^2)} \right] } ~\textrm{ml} \end{aligned}$$

where $A_{sag}$ is the bladder area in the sagittal plane and $A_{trans}$ is the bladder area in the transverse plane. Volume estimation was performed for ground truth clinician annotations and model outputs, and the results were compared.

### Evaluation

Each segmentation network is evaluated with three criteria: segmentation and clinical accuracy, memory requirement, and computation requirement.

We calculate the Dice scores for FP and B+FP versions of U-Net, DeepLabv3+, and Panoptic from the model output masks. Then, a B+Q version of the best-performing model is developed. Multiple versions of this model are compared to the clinician’s annotations using Bland-Altman plots, with the 95% confidence interval labeled. Since the clinician’s annotations are considered more reliable than the neural network models, the clinician’s volume estimate is used as the x-axis instead of the mean of both methods. Moreover, we used concordance correlation coefficients to analyze the agreement of the model to the clinical ground truth.

There are two types of memory requirements: the first consists of neural network parameters, which must be permanently stored on-chip. Past works have termed its reduction the model compression ratio^20^. The second type of memory requirement occurs only during inference, which has rarely been discussed in past works^5,18–20^. It is the memory required to store the intermediate feature maps for later use in the model. In the case of the U-Net in Supplementary Fig. S2, the four horizontal arrows, indicating the skip connections between the encoder and the decoder, represent this type of memory requirement.

We report the multiplies and accumulates (MACs) required for computation requirements. It is important to note that floating-point computation is much more energy and chip area intensive than binary computations. A binary MAC only needs a single XNOR followed by a count operation. For quantized MACs, we count the MAC of an *a*-bit number and a *b*-bit number as $a\times b$ binary MACs. Please refer to Supplementary Information for more details on how memory and computation requirements are obtained.

## Results and discussion

### Selection of algorithm

**Figure 3:**
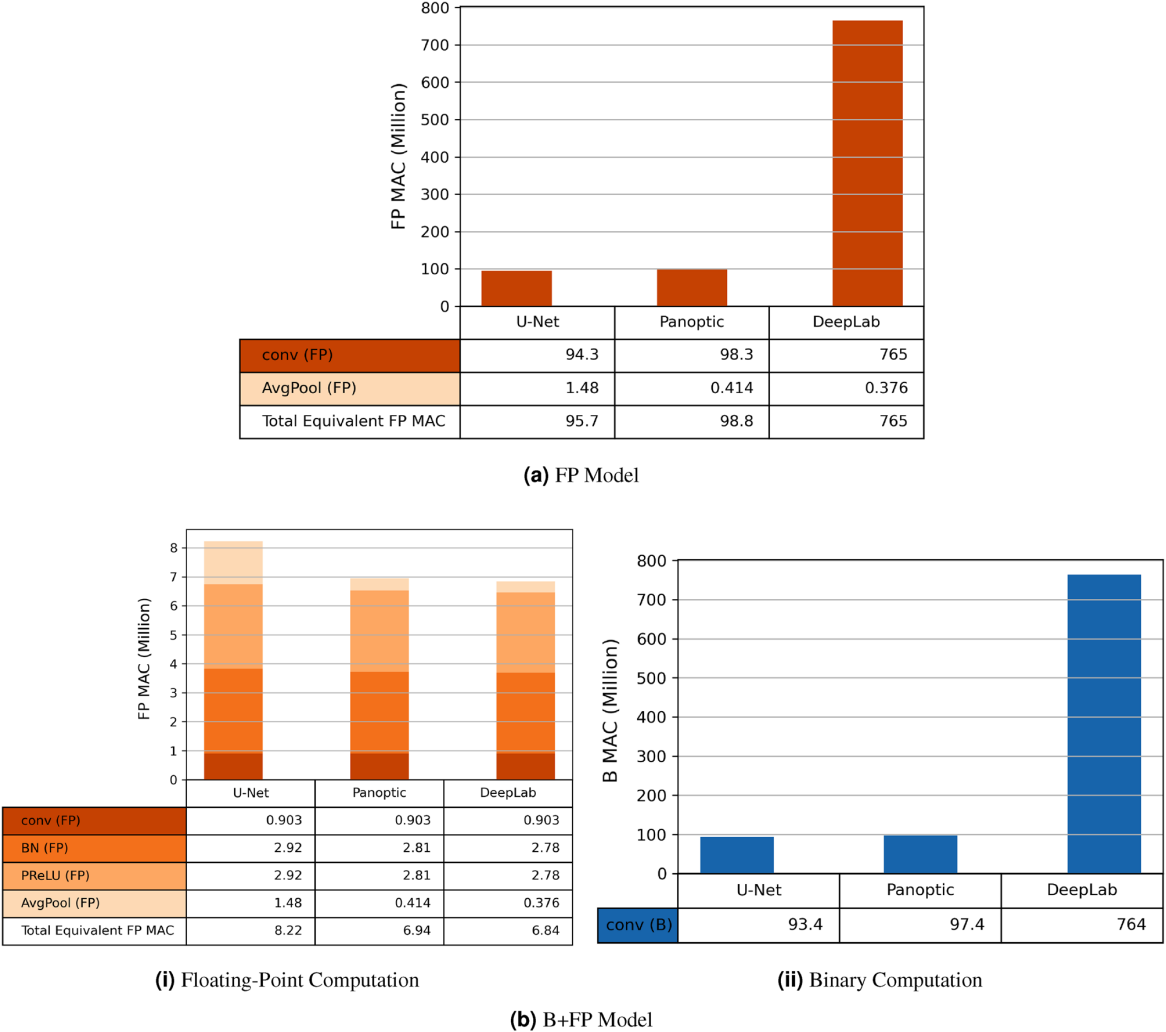
Computation requirement in the unit of a million MACs. The DeepLabv3+ Model requires more than $7\times$ computations than U-Net and Panoptic. Orange bars are used for floating-point MACs; blue bars indicate equivalent binary MACs. The models are defined in Table 1. Conv is convolution; AvgPool is average pooling; BN is BatchNorm; PReLU is parametrized ReLU [Eq. (3)].

Three criteria are used to select the optimal baseline model: accuracy in Table 2, memory requirement in Fig. 2, and computation requirement in Fig. 3.

In Table 2, the Dice scores of the 3 FP Models were within 1%, while U-Net performed best after binarizing most convolutional layers. Figure 3a,b showed that DeepLab was $8\times$ more computationally intensive than the other two alternatives. U-Net and Panoptic, compared in Fig. 3b, required similar amounts of binary and floating-point computations. Even though U-Net required more multiplication in BatchNorm and AvgPool, both can be replaced with binary shift operations in the B+Q Models. In the B+Q Model, shown in Fig. 2, U-Net B+FP Model required 240 kB and 288 kB less memory than the Panoptic and DeepLab B+FP Models, respectively. Since U-Net B+FP Model had the highest accuracy and needed the least memory and computation, it was chosen as the baseline. Its architecture is shown in Supplementary Fig. S2.

As shown in Fig. 2, binarization of the convolutional layers reduced the network parameter size, but not the skip connection size. In the case of the U-Net B+FP Model, the floating-point skip connections required $5.9\times$ memory than all of the model parameters combined. This motivated the development of the B+Q Model with quantized skip connections.

**Table 2:** Image segmentation Dice score.

Algorithm	FP model (%)	B+FP model (%)
U-Net	89.6	88.5
Panoptic	89.1	87.5
DeepLab	89.9	87.9

### Skip connection quantization and compression

Since the distribution after PReLU [Eq. (3)] was not symmetric with respect to 0, our experiments showed that the unsigned representation led to higher accuracy than the signed representation, using the same bit-width. The test accuracy with 6-bit, 4-bit, and 3-bit skip connections is 87.0%, 85.8%, and 71.8%, respectively. Increasing the bit-width beyond 6 bits increased the memory requirement linearly without significantly improving the accuracy. The 3-bit version was $14\%$ worse than the 4-bit version, rendering it unacceptable for clinical usage. Therefore, both models with 4-bit and 6-bit skip connection bit-width, i.e. B+Q (4*b*) Model and B+Q (6*b*) Model, were further investigated. More details of the memory and computation of the various B+Q U-Net Models and the B+FP baseline can be found in Supplementary Table S2.

Quantization limited the possible data values, lending itself well to compression. For instance, only $3.4\%$ of the values in the floating-point skip connections were zero, versus $38.3\%$ after the 6*b* quantization. Therefore, any compression technique that exploits the presence of zeros would be more efficient with the quantized data. Four lossless compression methods were applied to the quantized intermediate feature maps as described in the Methods section.

**Figure 4:**
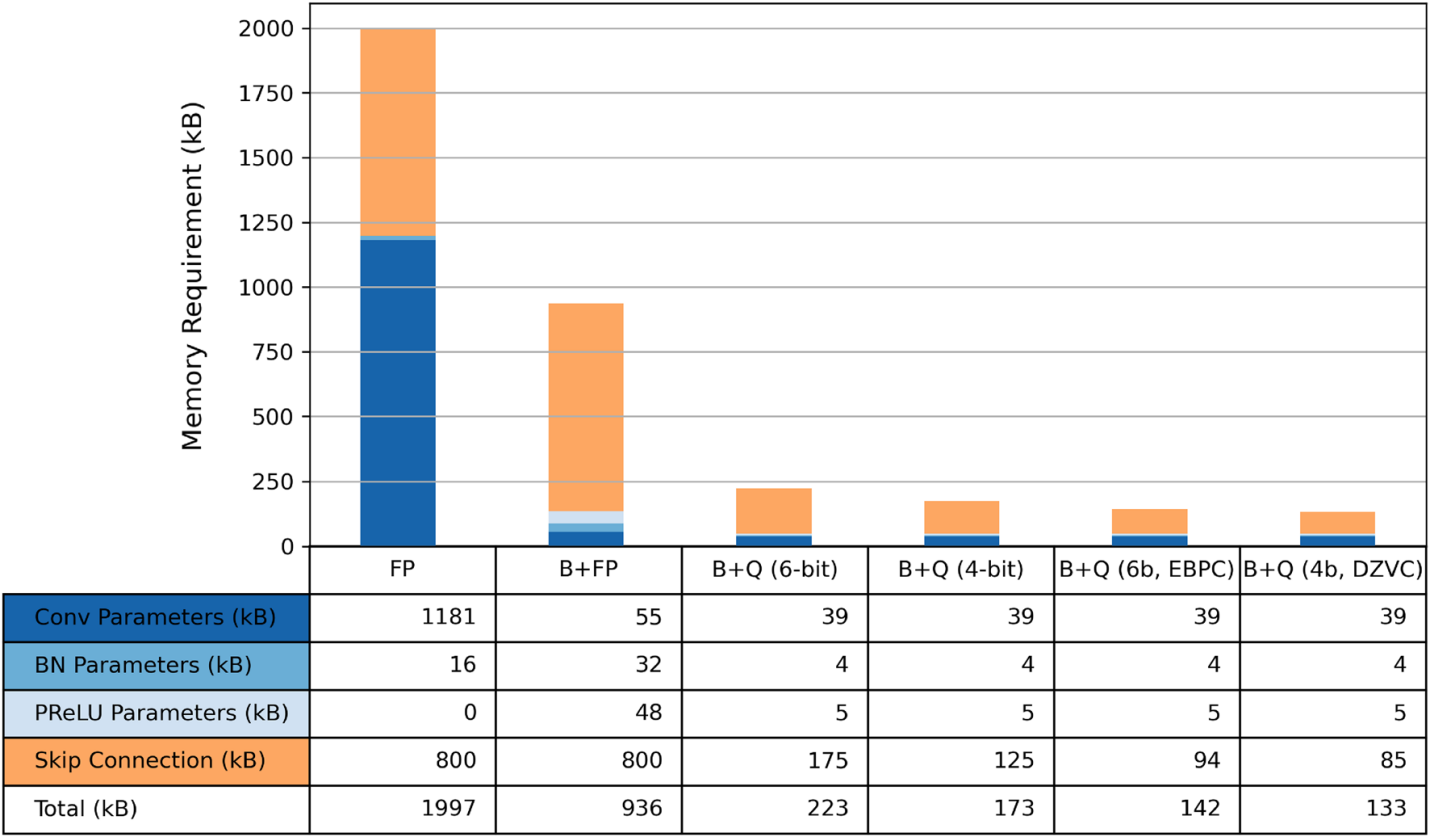
Memory requirement of FP, B+FP, and B+Q Models of U-Net. Blue bars indicate permanent memory requirement of model parameters; orange bars indicate additional memory requirement at inference. After applying the best out of 4 compression algorithm to B+Q Models (6*b* and 4*b*), the last two columns show that the skip connection memory is reduced by 46% and 32%, respectively.

The best compression technique for each model was plotted in Fig. 4. EBPC and DZVC offered similar efficiency for the B+Q (4*b*) Model, so DZVC was selected due to its more straightforward implementation. For the B+Q (6*b*) Model, EBPC offered the best compression and achieved a 36% storage reduction.

### Advantages of binarization, quantization and compression on ASIC design

The drastic reduction in memory requirement from 1997 kB of the FP Model to the 142 kB of the compressed B+Q (6*b*) Model translates directly to a $14\times$ reduction in memory area. In a binary NN accelerator, the memory typically dominates the chip area^21^. Binarization also significantly reduces energy cost. The energy cost of FP MAC (dominated by multiply operation) is around $100\times$ that of binary MAC (dominated by accumulation)^22^. While the algorithm runtime is heavily dependent on the target hardware platform, the reduced computation and memory access leads to a much higher segmentation frame rate. Interested readers are referred to our follow-up work for a $14.4\mu J$-per-inference real-time ASIC implementation of the compressed B+Q (4*b*) Model^21^.

### Effect of outer bladder wall annotation

The outer bladder wall annotation aided the NN to better extract feature information during backpropagation in the training process, thereby leading to significantly higher Dice scores. The Dice scores were improved by 4.5%, 3.3%, and 4.3% for the FP, B+FP, and B+Q Models respectively (details in Supplementary Table S3).

### Clinical evaluation

Figure 6 compared the bladder volume estimates of the NNs and the radiologists using Bland-Altman analysis. The mean volume estimation errors of the B+Q Model with 4-bit and 6-bit skip connections were $5.0\pm 33$ ml and $6.8\pm 29$ ml ($\text {mean}\pm \text {standard deviation}$). The full precision method performed better with an error of $2.6\pm 19$ ml, but at the cost of excessive computation and memory space. The biases of our models were below 7.3 ml, which were reasonable compared to the bias of $-$ 10 ml and $-$ 20 ml for men and women in Marks et al.^28^.

The concordance correlation coefficients (CCCs) of the FP Model and B+FP Model were 0.984 and 0.969. The B+Q (6*b*) Model, which eliminated the use of FP operations, presented minimal degradation from B+FP Model with 0.962 CCC. The B+Q (4*b*) Model had slight degradation in volume estimation at a CCC of 0.949.

**Figure 5:**
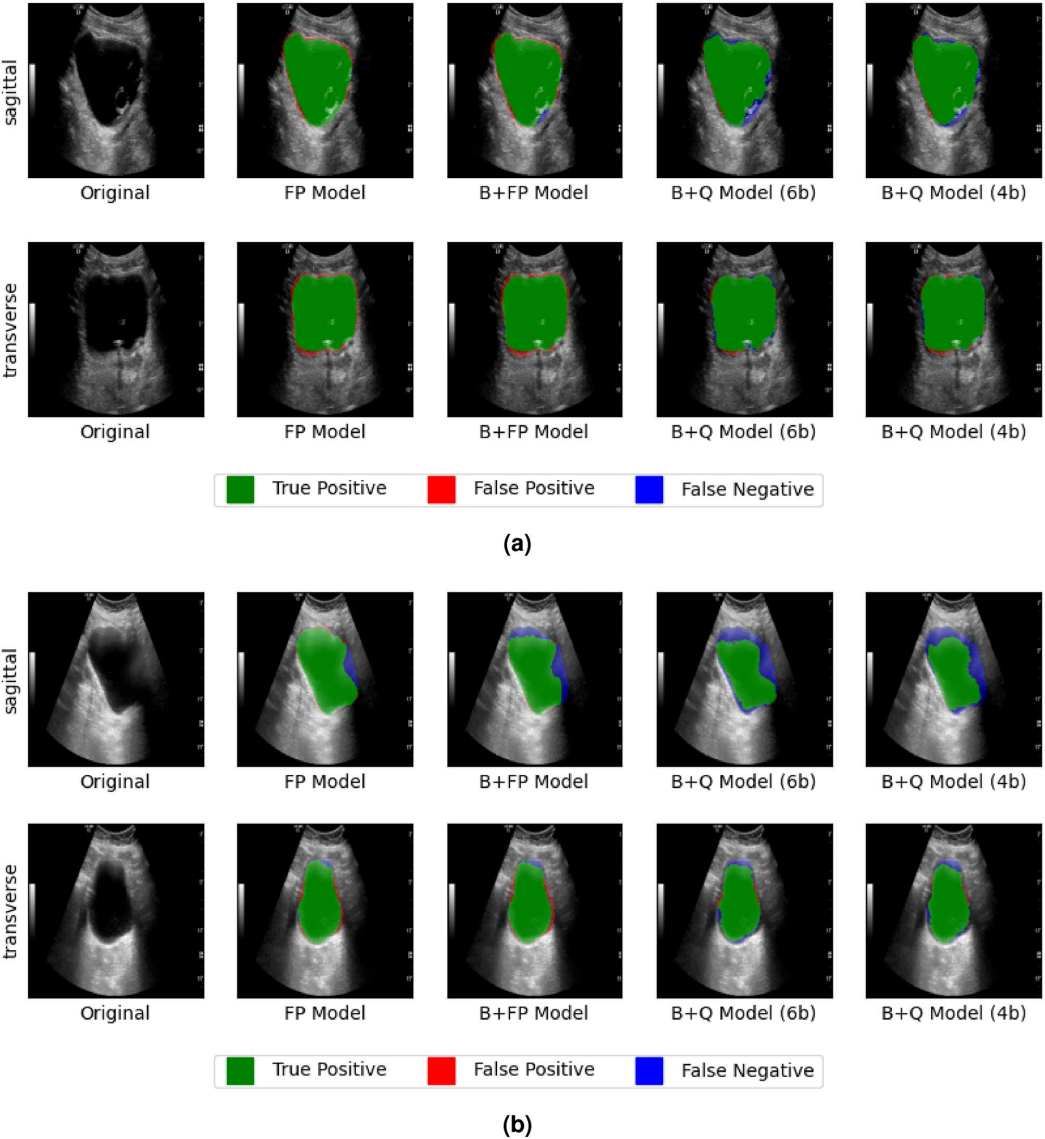
(**a**) An example bladder image showcasing the algorithms ability to estimate the bladder walls correctly despite the presence of a Foley catheter. (**b**) An example case with poorly defined bladder wall on the right hand side, leading to poor segmentation results.

An example of the segmentation result was shown in Fig. 5a. In this image, the algorithm was able to segment the bladder inner wall properly despite the presence of a Foley catheter inside the bladder. Figure 5b showed an example of poorly defined bladder wall on the right side. The poorly defined wall led to segmentation error which was propagated in estimating bladder volume.

**Figure 6:**
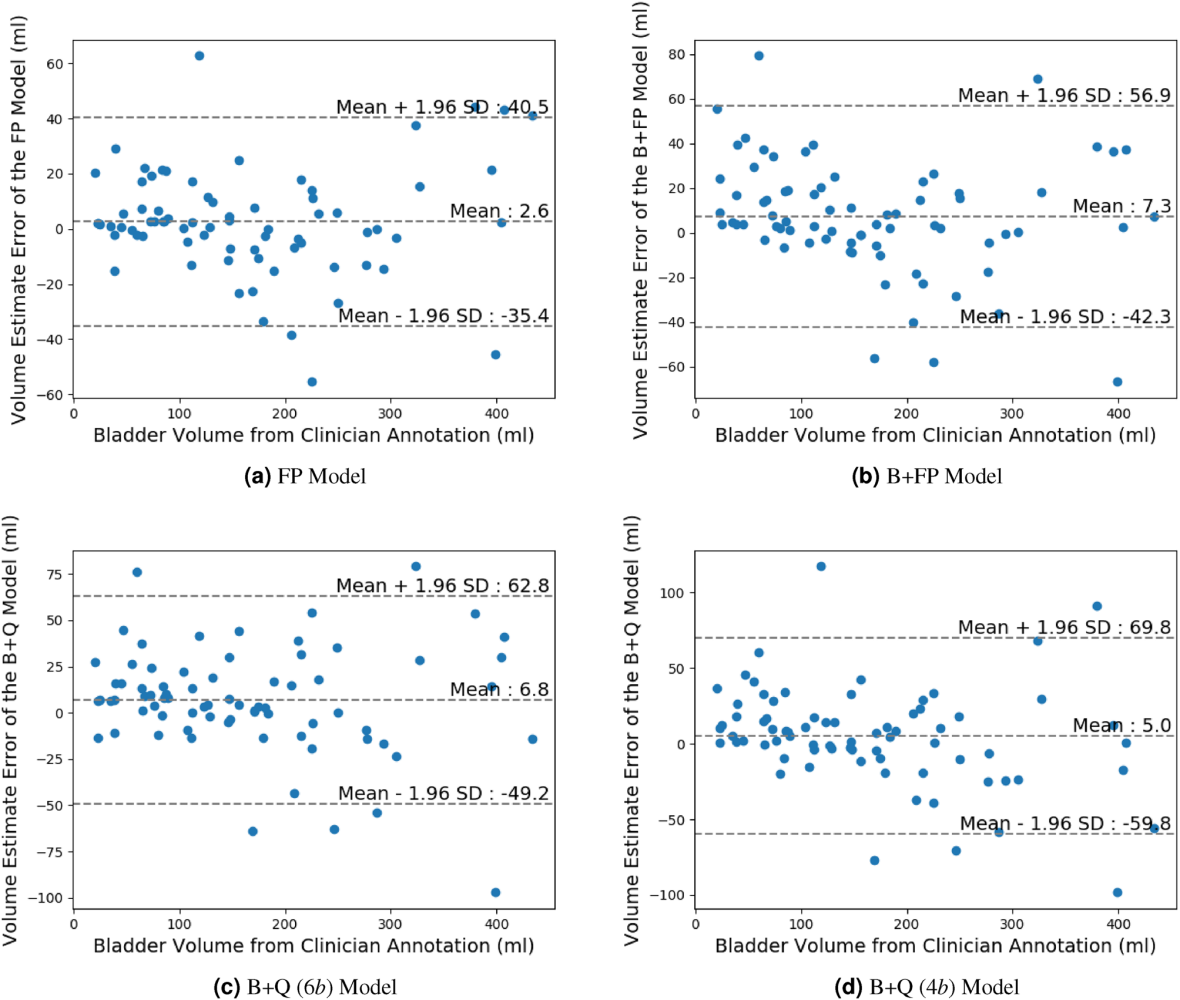
Bland-Altman plots of the downstream volume estimation task. Since clinician annotation is considered the ground truth, it is used as the x-axis, instead of the average of the clinician annotation and the U-Net model estimate. The volume estimates in (**a**) (**b**) (**c**) (**d**) use the segmentation outputs of the FP, B+FP, and B+Q (6*b*, 4*b*) Models, respectively. The middle horizontal lines represent the average difference, i.e. bias, in volume estimation between our segmentation approach and clinician segmentation. The upper lines and lower lines indicate the 95% confidence interval of mean difference.

### Future work and applications

While unsupervised learning enables the use of a larger dataset, it is an emerging, but unproven technique in ultrasound imaging^29^. A potential direction is to apply existing unsupervised learning techniques from other domains to ultrasound^30^. The same quantization and compression techniques can be applied to the NN to reduce memory and computation, regardless of the training procedure.

This paper’s binarization and compression techniques can be applied to other medical applications. For instance, Ouyang et al.^31^ implemented a lightweight NN for seizure classification on the neuromodulation implant device. However, there are larger NNs that can provide more detailed seizure classifications, which were not implemented on the device due to computation and memory constraints^31^. With our algorithm technique to reduce memory and computation requirements, we can utilize more sophisticated NNs on biomedical devices.

## Conclusion

We developed and evaluated a low-memory and low-compute deep-learning model for bladder segmentation and urine volume estimation. Compared to prior works^5,18–20^, we eliminated the need for floating-point computations and reduced the memory requirement to fit on low-cost microcontrollers (detailed comparison in Supplementary Table S1). With skip connection quantization, the B+Q (4*b*) and B+Q (6*b*) Models reduced the total memory requirement by $11.5\times$ or $9.0\times$; using DZVC for B+Q (4*b*), the total memory was further reduced by $23\%$; using EBPC for B+Q (6*b*), $36\%$ of the memory requirement was reduced. The reduction of RAM requirement from 800 to 85 kB or 94 kB makes the algorithm friendlier to deploy on edge devices, such as area-constrained low-cost ASICs. The accuracy of the B+Q (4*b*) and B+Q (6*b*) Models was within $3.8\%$ and $2.6\%$ of the state-of-the-art segmentation models that required hundreds of millions of floating-point operations and were much more memory-intensive than our proposed model. Bladder volumes calculated from our models were on par with volumes from ground-truth clinician annotations with CCCs of 0.962 and 0.949. The availability of low-compute, low-memory, accurate, and automated bladder volume monitoring algorithm will enable the development of low-cost smart ultrasound devices, which will help diagnose POUR in its early stage and prevent CAUTI by reducing catheterization. Using continuous bladder volume monitoring as the case study, the same compression and quantization techniques can be applied to other image segmentation models that would be beneficial to deploy on computation and memory constrained wearable medical devices.

### Supplementary Information

Supplementary Information.

## Data Availability

The anonymized data is available for scientific purpose upon reasonable request to S.S. (sschoenjr@mgh.harvard.edu) pending the IRB review and approval, and applicable data use agreements.
