# Supplementary material for: Memory-efficient low-compute segmentation algorithms for bladder-monitoring smart ultrasound devices
Source: Sci Rep. 2023 Sep 30;13:16450. doi: 10.1038/s41598-023-42000-9 (PMC10542811; doi:10.1038/s41598-023-42000-9)
Supplement: Supplementary file 1 — Supplementary Information. [file 41598_2023_42000_MOESM1_ESM.pdf]

Supplementary Information for *Memory-efficient low-compute segmentation algorithms for edge bladder monitoring ultrasound devices*

by Zhiye Song, Mercy Asiedu, Shuhang Wang, Qian Li, Arinc Ozturk, Vipasha Mittal, Scott Schoen Jr., Srinath Ramaswamy, Theodore T. Pierce, Anthony E. Samir, Yonina C. Eldar, Anantha Chandrakasan, Viksit Kumar

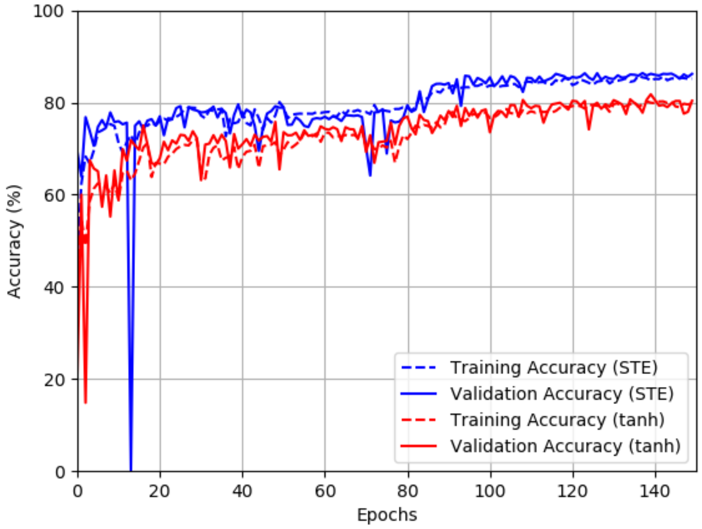

Figure 1. Training curves of the Binary + Quantized Model with 4-bit skip connections (B+Q (4*b*) Model). The model trained with Straight-Through Estimator (STE) converged to a better accuracy than with tanh.

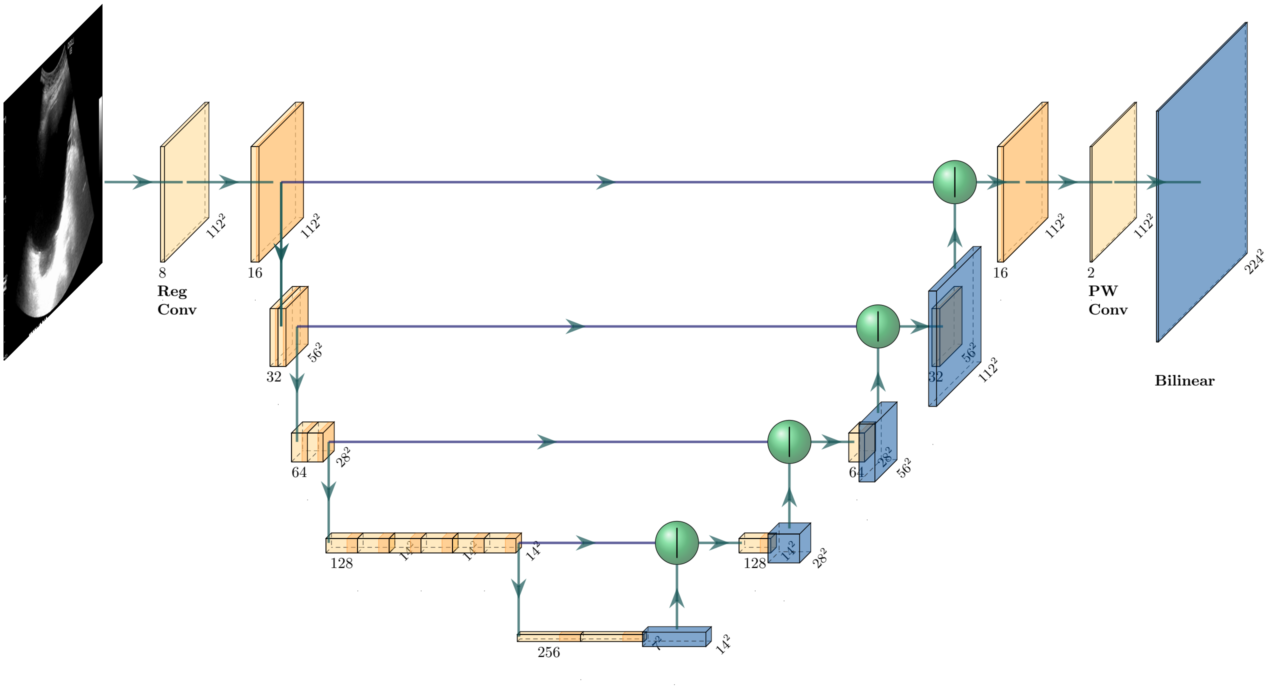

Figure 2. U-Net Architecture. **Reg Conv** stands for regular convolution; **PW Conv** stands for pointwise convolution. All yellow banded boxes stand for depthwise-separable convolutions. The last convolutional layer at each resolution level in the encoder branch has a stride of 2. **Bilinear** stands for a bilinear upsampling; the remaining blue boxes stand for upsampling layers with the nearest neighbor mode. The | symbol in the green circle stands for concatenation.

	TernaryNet[1]	U-Net fixed-point[2]	MedQ[3]	Previous work[4]	This work
Most Conv (convolutional) Layers	Ternary	4-bit weight 6-bit input	Ternary/Binary	Binary	Binary
First Conv Layer	FP	FP	FP	FP	4-bit
Last Conv Layer	Ternary	FP	FP	Binary	Binary
Skip Connections	Ternary	6-bit	FP (including ResNet-style connections)	FP (including ResNet-style connections)	4-bit/6-bit
Scaling Factor	FP	-	-	FP	-
BatchNorm	FP	Fused	FP	FP	Binary shift
Activation Function	Quantization function	ReLU	ReLU	Parametrized ReLU with FP	Parametrized ReLU with 2-bit
Accuracy Loss compared to FP	4.7%	2%	1% / 3%	within 3%	3.8%/2.6%

Table 1. Comparison to other quantized neural network models in medical image segmentation (FP stands for floating-point).

U-Net Models	B+FP	B+Q (6*b*)	B+Q (4*b*)	B+Q (3*b*)
Test Accuracy	88.5%	87.0%	85.8%	71.8%
Memory Requirement of Skip Connections	800kB	175kB	125kB	100kB
Computation Requirement (Million of FP MACs)	8.22	0		
Computation Requirement (Million of B MACs)	93.4	201		

Table 2. U-Net Binary+Floating-Point (B+FP) and Binary+Quantized (B+Q) Models with different bit-widths of the skip connections as shown in the parenthesis.

**Annotations**	U-Net (FP Model)	U-Net (B+FP Model)	U-Net (B+Q (4*b*) Model)
Inner and outer bladder walls	89.6%	88.5%	85.8%
Inner bladder wall only	85.1%	85.2%	81.5%

Table 3. Model performance (Dice Score) with and without outer bladder wall annotations.

Memory requirement calculation

For each skip connection, i.e. each horizontal line in Supplementary Figure 2, two different formats are stored in memory. The first is the binary feature map of size *number of channels* $\times$ *feature map width* $\times$ *feature map height* bits. The second is the quantized feature map. In the B+Q (6*b*) Model, for example, each skip connection size is *number of channels* $\times$ *feature map width* $\times$ *feature map height* $\times$ 6 bits. Note that the binary feature map cannot be derived from the quantized feature map and the threshold parameter α, because the stored quantized feature map has a lower precision than the intermediate computation results.

Computation requirement calculation

For a convolution layer, the number of MACs is *input channels* $\times$ *output channels* $\times$ *kernal height* $\times$ *kernal width* $\times$ *output height* $\times$ *output weight*/*groups*. The depthwise separable structure reduces the number of MACs, because the number of groups is increased from 1 to the number of input channels in a depthwise layer, and kernal size is reduced from 3×3 to 1×1 in a pointwise layer. For the BatchNorm layer, the FP Model does not require additional MAC since it can be fused into the convoluion. The other models require a number of MAC equal to the input tensor size. For the activation layer, the FP Model does not require additional MAC since it is a simple ReLU layer. Since the activation function PReLU operates on each input value, the MAC is equivalent to the input tensor size for quantized models. For average pool layers, the MAC is also equivalent to the input tensor size, since each input value is accumulated and divided.

References

1. Heinrich, M. P., Blendowski, M. & Oktay, O. TernaryNet: faster deep model inference without GPUs for medical 3D segmentation using sparse and binary convolutions. *Int. J. Comput. Assist. Radiol. Surg.* **13**, 1311–1320, DOI: 10.1007/s11548-018-1797-4 (2018).

2. AskariHemmat, M. H. *et al.* U-net fixed-point quantization for medical image segmentation. *Med. Imaging Comput. Assist. Interv. (MICCAI), Hardw. Aware Learn. Work. (HAL-MICCAI) 2019* **11851 LNCS**, 115–124, DOI: 10.1007/978-3-030-33642-4_13 (2019).

3. Zhang, R. & Chung, A. C. MedQ: Lossless ultra-low-bit neural network quantization for medical image segmentation. *Med. Image Analysis* **73**, 102200, DOI: 10.1016/j.media.2021.102200 (2021).

4. Brahma, K., Kumar, V., Samir, A. E., Chandrakasan, A. P. & Eldar, Y. C. Efficient Binary CNN For Medical Image Segmentation. In *2021 IEEE 18th International Symposium on Biomedical Imaging (ISBI)*, 817–821, DOI: 10.1109/ISBI48211.2021.9433901 (2021).
